# Construction and validation of a tool to Assess the Use of Light Technologies at Intensive Care Units**^1^**

**DOI:** 10.1590/1518-8345.1002.2816

**Published:** 2016-12-19

**Authors:** Pabliane Matias Lordelo Marinho, Maria Pontes de Aguiar Campos, Eliana Ofélia Llapa Rodrigues, Cristiane Franca Lisboa Gois, Ikaro Daniel de Carvalho Barreto

**Affiliations:** 2 Specialist in Health Administration, RN, Hospital Universitário, Universidade Federal de Sergipe, Aracaju, SE, Brazil.; 3 PhD, Associate Professor, Departamento de Enfermagem, Universidade Federal de Sergipe, Aracaju, SE, Brazil.; 4 PhD, Adjunct Professor, Departamento de Enfermagem, Universidade Federal de Sergipe, Aracaju, SE, Brazil.; 5 Statistics.

**Keywords:** Biomedical Technology, Nursing, Intensive Care Units, Validation Studies

## Abstract

**Objective:**

to construct and validate a tool to assess the use of light technologies by the nursing team at Intensive Care Units.

**Method:**

methodological study in which the tool was elaborated by means of the psychometric method for construction based on the categorization of health technologies by Merhy and Franco, from the National Humanization Policy, using the Nursing Intervention Classification taxonomy to categorize the domains of the tool. Agreement Percentages and Content Validity Indices were used for the purpose of validation.

**Results:**

The result of the application of the Interrater Agreement Percentage exceeded the recommended level of 80%, highlighting the relevance for the proposed theme in the assessment, with an agreement rate of 99%.

**Conclusion:**

the tool was validated with four domains (Bond, Autonomy, Welcoming and Management) and nineteen items that assess the use of light technologies at Intensive Care Units.

## Introduction

Light Technologies (LT) are important to promote care humanization, as they emphasize the relation between the professional and the patient, patient-centered listening and satisfaction of the patient's needs^1^ ^-^ ^2^. Their insertion in care influences the care practice through the selection of care models that strengthen and qualify the Nursing work process^3^. As a result, the literature refers to the valuation of clinical reasoning for the elaboration of the individualized care plan^4^.

Due to their relational focus, these technologies are part of the guiding values of the National Humanization Policy (NHP) ^5^ launched in 2003. The aim of the policy is to produce autonomous subjects who are protagonists and co-accountable for the health production process, determining a set of actions that promote transformations in the relationship and communication modes among the subjects^6^. 

To elaborate an assessment tool of LT, the Nursing Intervention Classification (NIC) was chosen, due to its applicability in different care areas, range, clinically significant language and up-to-dateness for clinical practice and research^7^. In Brazil, the NIC has been applied at different services, such as rooming-in, emergency, primary health care and surgery^8^. On the other hand, no precedent has been found for the use of NIC to support the measuring of light technologies at Intensive Care Units (ICU). 

In that sense, the assessment of health technologies is aimed at analyzing their implications, cost, dissemination and use, as well at knowing its effects on human life, work and society. It is very important to weight the consequences of technology use in the short, medium and long term and to support the health managers' political and clinical decision making^9^. 

The construction and validation of research tools has been frequently used as a method, as many nursing professionals have perceived the need to discuss these contents and constructs applied in daily practice, considering that there are not always tools available to faithfully measure these events^10^.

Hence, validating a research tool is a method to analyze the exactness of a certain inference elaborated based on test scores, it is more than the expression of the score of a measuring tool. It is an investigation that permeates the entire process, ranging from the elaboration to the application, correction and interpretation of the results. Validating the content means investigated whether it responds to all aspects of its object, observing its content and relevance of the objectives it serves to measure^11^.

The lack of a tool in the literature to assess the use of LT not only encouraged this study, but also makes it original, as it promotes the valuation and stimulus of humanization in care^1^ ^-^ ^2^ and the assessment of health technologies through strictly elaborated and validated tools^10^ ^-^ ^11^.

Hence, the objective in this study is to present a tool that was constructed and validated to assess the nursing team' use of light technologies at Intensive Care Units (ICU), as these steps are important to enhance its reliability^11^.

## Method

In this research, the methodological development of a tool to assess the use of LT by the ICU nursing team. First, using the psychometric method to construct a tool^12^, the semantic step was undertaken, based on Merhy and Franco's theory on LT and described in the NHP. Words were surveyed that had the same meaning and repeated in the concepts of the abovementioned authors. Next, the correspondence was established with the Nursing Interventions (NI) in NIC that best represented the identified points^7^.

As a result, 19 items were extracted that were grouped in the selected dimensions (Bonding, Autonomy, Welcoming and Management) and their use corresponded to the application of the LT. The name of the NI, its definition and numerical identification code were maintained. Individualizing care, the NIC allow the professional to choose the activities of each intervention according to what (s)he considers more suitable for each patient^7^.

Each NI consists of a list of activities the nurse selects to comply with the individualized care plan, without establishing a minimum activity criterion for each plan. Each of these activities considers the taime needed to make the intervention, defined in minutes, and the minimum educational background needed for a safe implementation, that is it defines actions for each team member^7^.

Thus, in the Bond domain, the following NI were established^7^: 1. Active Listening (4920) / 2. Touch (5460) / 3. Complex Relationship Building (5000) / 4. Security Enhancement (5380) / 5. Presence (5340). In the Autonomy domain: 6. Values Clarification (5480) / 7. Self-care Assistance (1800) / 8. Assertiveness Training (4340) / 9. Self-awareness Enhancement (5390) / 10. Emotional Support (5270).

In the Welcoming domain: 11. Admission Care (7310) / 12. Case Management (7320) / 13. Surveillance: Safety (6654) / 14. Shift Report (8140) / 15. Visitation Facilitation (7560). And in the Management domain: 16. Critical Path Development (7640) / 17. Decision-making Support (5250) / 18. Health Literacy Enhancement (5515) / 19. Patient Rights Protection (7460).

After the semantic phase, the second step of the validation method was undertaken. This was based on the content validation using the Interrater Agreement Percentage and the Content Validity Index (CVI). Validation is important because, after developing the concept and formulating its dimensions, the researcher submits it to a group of raters who are considered experts in the area, considering that the content validation is judgment-based^13^.

Six judges were selected based on the criteria adapted from Fehring^14^: being an expert in Health Technologies; being a Nurse Faculty active in ICU; working as a Clinical Nurse in ICU for about 10 years; scoring on the thematic Intensive Care/Health Technology items: Dissertation (02 points); Thesis (02 points); practical experience / (02 points); participation in research group/project (01 point) and authorship or co-authorship of paper published in journals (01 point per paper, maximum 10 points).

The raters judged each item on the form according to the following six criteria^11^: Face: criterion attributed to the aspect, form and external side of the form; Clarity/understanding: links transparency, perceptibility and understandability of the data; Content: refers to the content of each item; Efficiency/Consistency: refers to the production of a desired effect or a good result associated with the reality, veracity and firmness of the data; Objectivity: criterion attributed to the observation of the question itself. Understandable without mixing personal ideas; and Validity for the proposed model: this criterion refers to the adaptation and suitability of the tool to the LT concepts of Merhy and Franco^1^, the NHP^5^ and the NIC Nursing Interventions^7^.

The assessments were forwarded to the experts electronically, accompanied by their respective description. Only one of the following options could be marked: "Appropriate", "Needs adaptation" and "Inappropriate (modified on a Likert scale) for each assessment criterion of the tool. In the first analysis, space was provided for comments and suggestions. After finishing the electronic completion, the answer was forwarded automatically. No inclusions or exclusions of Nursing Interventions in the tool were suggested.

The answers were scored as follows: "Appropriate" = 0, "Needs Adaptation" = 1 and "Inappropriate" = 2. To assess the content validity, the Percentage of Agreement was used. To be considered satisfactory, the minimum percentage of interrater agreement was set at 80%^13^.

Next, the CVI was applied to measure the proportion or percentage of interrater agreement on the items^15^, whose item rate or score should be superior to 0.78^13^ ^,^ ^15^.

Approval for the study was obtained from the Ethics Committee at Universidade Federal de Sergipe, protocol 875.505, in compliance with National Health Council Resolution 466/12, and is part of the Master's Thesis in Nursing "Use of Light Technologies by the Nursing Team at Intensive Care Units: a comparative study". All participants signed the Informed Consent Form.

## Results

The tool consisted of four domains: Bond, Autonomy, Welcoming and Management. Each domain was described by at least four and at most five Nursing Interventions with their respective identification codes. No activities were specified due to the freedom the NIC itself grants the nurse to select them according to hers and the patient's reality. Hence, the tool was forwarded with 19 elaborated items for the purpose of the first content validation by the raters.

The raters' ages ranged between 28 and 56 years (mean 40 years and SD ± 9.7), five of them being women (83.3%). Five raters were knowledgeable on Intensive Care (13 to 25 years), three of whom also possessed experience in Health Technologies (06 to 12 years) and one rater was only knowledgeable on Health Technologies (06 years). Four held a Ph.D. and one a post-Ph.D. Five were proficient in at least one foreign language (English, Spanish or French). Hence, in the thematic items (Dissertation, Thesis, practical experience, participation in research group, authorship or co-authorship of studies published in journals) obtained a mean score of 6 points.

The experts always gave suggestions related to the title of the item and the harmonization of the writing style; however, as the language had been standardized by the researchers from the University of Iowa, these could not be modified.

**Figure 1:**
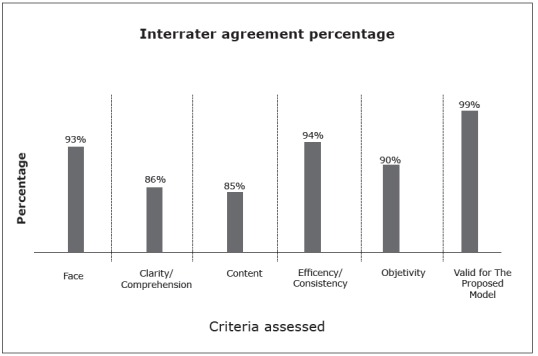
Interrater Agreement Percentage per criterion, Aracaju, SE, Brazil, 2015

The result of the application of the Interrater Agreement Percentage exceeded the recommended level of 80% (Figure 1), highlighting the relevance for the proposed theme in the assessment, with an agreement rate of 99%.

The items were assessed individually using the CVI and grouped in four domains according to the classification of the proposed model (Bond, Autonomy, Welcoming and Management). The mean CVI in the first analysis corresponded to 0.90; 0.88; 0.89 and 0.93, respectively and, in the second analysis, 0.93; 0.91; 0.91 and 0.93, that is, always higher than the predetermined CVI ≥ 0.78.

**Figure 2:**
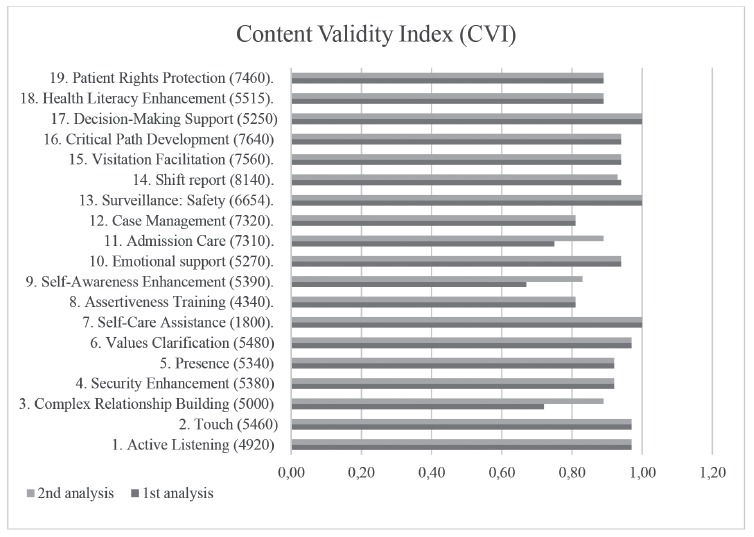
Content Validation Index (CVI) per item. Aracaju, SE, Brazil, 2015

In the first analysis, three items presented a CVI inferior to 0.78: a) Complex Relationship Building (CVI = 0.72), b) Self-awareness Enhancement (CVI = 0.67) and c) Admission Care (CVI = 0.75), after the re-evaluation by the experts, the indices changed to: a) Complex Relationship Building (CVI = 0.89), b) Self-awareness Enhancement (CVI = 0.83) and c) Admission Care (CVI = 0.89) (Figure 2).

The general CVI of the tool constructed after the two analyses was 0.92, ranging between 0.83 and 1.00 among the Nursing Interventions; 0.91 to 0.93 among the domains, that is, always superior to the 0.78 recommended in the literature.

## Discussion

The LT propose work processes that value the relation between the patient and the nursing professional^3^ ^-^ ^4^. The ICU patients' profile at this moment demands specific care processes, considering that, in most cases, the care is intermediated by devices and equipment and the relation becomes distant and impersonal^16^. Hence, reflecting on and analyzing the construction and validation of a tool to assess the use of LT makes this study relevant and original, in view of the growing importance of humanization and patient safety. 

The use of LT improves healthcare, due to the acknowledgement of the psychological and social, emotional and spiritual aspects, valuing the human being and communication, and therefore establishing an affective relation between nursing professional and patient and implementing welcoming actions, with a view to the patient's autonomy. Thus, the care management promotes the security and guarantees the patient's right to error-free care^1^.

Hence, the construction and validation provides for a tool that is intended to faithfully measure its object^17^, in this case LT, and will support the professionals to effectively implement it in their care processes.

A group of six experts performed the content validation, a number considered sufficient for the process^12^ ^-^ ^13^ ^,^ ^17^. In the first analysis, the lowest interrater agreement percentage was found for the item clarity/content (77%). Although the judges recommended improving the language, after forwarding an explanatory note on the impossibility of changes due to the standardized language of the NIC, the NI were accepted and their continuation in the tool was confirmed, increasing to 86%, superior to the recommended rate^12^.

The approximation between the NI and LT, in view of the intrinsic relation between them and the care nursing professionals provide, strengthened the importance of using a standardized language like the NIC to categorize the content by means of a specific terminology^7^. 

Despite the difficulty the judges reported, the advantage of using the NIC lies in the possibility to broaden the professional's choice to certain activities, thus emphasizing the individual nature of care and the patient's participation in the conduction of the proposed care plan, in the care as well as the educational aspect^7^.

The results found suggested the interrater reliability. Nevertheless, it should be highlighted that the agreement is not a fixed property of measuring tools. To impede interference of the application context, the judges need to be sure on the concepts, descriptors and evaluation criteria^12^ ^-^ ^14^.

It is highlighted that the nurse experts with experience in teaching, research, care and management played an essential role in the validation process of the tool, and the use of Fehring's adapted method demonstrates the professionals' growing interest in the theme technologies involved in the work processes and certifies the expertise on the constructs discussed in this study. In that sense, recruiting professionals with longer experience in the area guarantees greater accuracy in the selection and evaluation of the tool^14^.

Concerning the assessment using the CVI, the domains of the tool always scored higher than the predetermined CVI ≥ 0.78. The overall CVI equaled 0.92. With 0.93, the domain Bond presents the NU that should be applied to strengthen the relation between nursing professional and patient. In line with Brazilian and international studies, nursing care is an interactive, reciprocal and interpersonal process and the professional's availability and physical interaction reveals the quality of the care, that is, therapeutic touch, kindness and other actions permit interaction and the establishment of a therapeutic and complex relationship^6^ ^,^ ^16^ ^,^ ^18^ ^-^ ^19^.

The ICU nursing professionals' difficulty to bond with severe patients is certified, especially when the patient uses an orotracheal tube or is sedated or when routine care like position change is provided, besides the mechanization of care, accelerated by the intense contact with equipment and devices, and the acknowledgement of interaction as care technology^3^ ^,^ ^19^.

The Autonomy domain shows how the NI are related with actions that enable the patients to create their own rules of functioning when they are under intensive care, helping them to understand and cope with the situation experienced. The care provided should offer emotional and physical support, mainly for self-care, despite the ICU patients' impossibility^16^ ^,^ ^18^ ^-^ ^19^.

Due to their disease and severity, the lack of familiarity with the equipment and devices, the routines and the team, the patients' vulnerable profile turns the ICU into a hostile environment for patients, relatives and companions. The service routine makes it a stressful environment with great risks for patient safety. This situation produces conflicting feelings in the nursing professionals, who need to respond to psychological, spiritual and social demands but are not trained to solve these situations. It is the role of the daily activities to solve the gap between theory and practice^16^.

The Welcoming domain considers the NI that refer to the nursing professionals' full accountability for the patient, with a view to guaranteeing a problem-solving and continuing care, close to the reference persons in their social life. Hence, welcoming involves information collection and exchange actions that improve the quality of health care and achieve the desired outcomes. In addition, it approaches the relatives and clarified the situations the patients experience, mainly the flexibilization of visiting times^16^ ^,^ ^20^.

This welcoming, problem-solving and human care responds to the reality many professionals experience, due to the unsatisfactory and unhealthy work conditions, which should be assessed to reflect on the work conditions and how this can influence the humanized care and the application of the LT^16^ ^,^ ^21^.

The Management domain show how NI remodel the ways the work processes are managed, including the patients and nursing professionals' greater involvement in care planning and in the defense of the patients' rights. At the ICU, beyond all care and management functions, the professionals should serve as educating agents. In this situation, education should be focused on enabling the patients to be able to process and understand the health-related information, thus supporting them to make decisions on their care, taking on a dialogical posture of respect for the severe patient's values^16^.

Management actions focused on the application of LT produce professional satisfaction and motivation for increasingly humanizing actions and the society that needs care from nursing professionals has already noticed this fact, mainly in ICU, making the care non-technical. It also grants the managers possible strategies to implement the NHP and LT^16^ ^,^ ^21^, especially inside the ICU.

This tool was elaborated and validated for application in a Master's thesis, as there are no Brazilian or international studies that address this gap, that is, that permit the assessment of LT use in ICU.

## Conclusion

Based on this study, it can be concluded that the tool represents the content on LT, and is therefore capable of measuring its use by the nursing team. Thus, the barrier related to the proposal of a tool was overcome.

In addition, the experts effectively participated in the validation of the tool and in proving the representativeness of the proposed model.

The tool should be reapplied in other care contexts than the ICU, in view of its contribution to strengthen the implementation of the NHP and to care centered on human beings.
